# Neuroendocrine Neoplasms of the Head and Neck: A Case Report on an Oropharyngeal Presentation

**DOI:** 10.7759/cureus.39823

**Published:** 2023-06-01

**Authors:** Josep Maeso Riera, Xavier Tarroch Sarasa, Javier Lao Luque, Laura Palomino Meneses

**Affiliations:** 1 Otolaryngology, Head and Neck Surgery, Sanitas CIMA Hospital, Barcelona, ESP; 2 Otolaryngology, Head and Neck Surgery, Hospital Universitari Mutua Terrassa, Terrassa, ESP; 3 Pathology, Hospital Universitari Mutua Terrassa, Terrassa, ESP

**Keywords:** surgery, treatment, histochemical markers, diagnosis, second tumor, oropharynx, neuroendocrine tumor

## Abstract

Neuroendocrine neoplasms (NENs) are a heterogeneous group of tumors that can appear in many different locations as neuroendocrine cells are distributed throughout the anatomy during embryonic development. This paper presents a case report of a 77-year-old woman with a rare NEN in the lateral wall of the pharynx. In addition to being very infrequent, it can be considered a second metachronous tumor since it is unrelated to a previous sinonasal NEN that the patient presented with 20 years before. We have reviewed the histological characteristics of NENs and the grading system used to determine their potential for metastasis or local invasion. NENs in the oropharynx are very infrequent and typically do not present systemic symptoms or specific local signs. The article concludes that surgical excision is typically the preferred treatment for localized NENs where complete removal is possible.

## Introduction

The neuroendocrine neoplasms (NENs) designation includes a diverse group of epithelial-like tumors arising from the diffuse neuroendocrine system. Although the pancreas, lungs, and digestive tract are the most common locations, NENs can appear in many different locations since these cells are widely distributed throughout the body during embryonic development [1, 2]. They are characterized by the capacity to produce a series of molecules with hormonal functions that can lead to the presentation of systemic symptoms. Most of these neoplasms are sporadic, not hereditary, and no known risk factors exist. However, some cases may be associated with hereditary syndromes, such as multiple endocrine neoplasms (MEN types 1 and 2). Histologically they present as small cell tumors with scant cytoplasm and characteristic immunohistochemical markers of neuroendocrine cells, the most relevant chromogranin and synaptophysin. The Ki67 nuclear proliferation marker index will determine the degree of potential replication of the tumor [3]. The presentation in the head and neck area is rare, particularly in the oropharynx, where only 52 cases have been identified up to the year 2021 [4]. This could be the first case of NEN presenting as a second tumor in the head and neck area in the literature. The patient has given informed written consent for using information and images of her case, with prior erasing of all personal identification data.

## Case presentation

**Figure 1:**
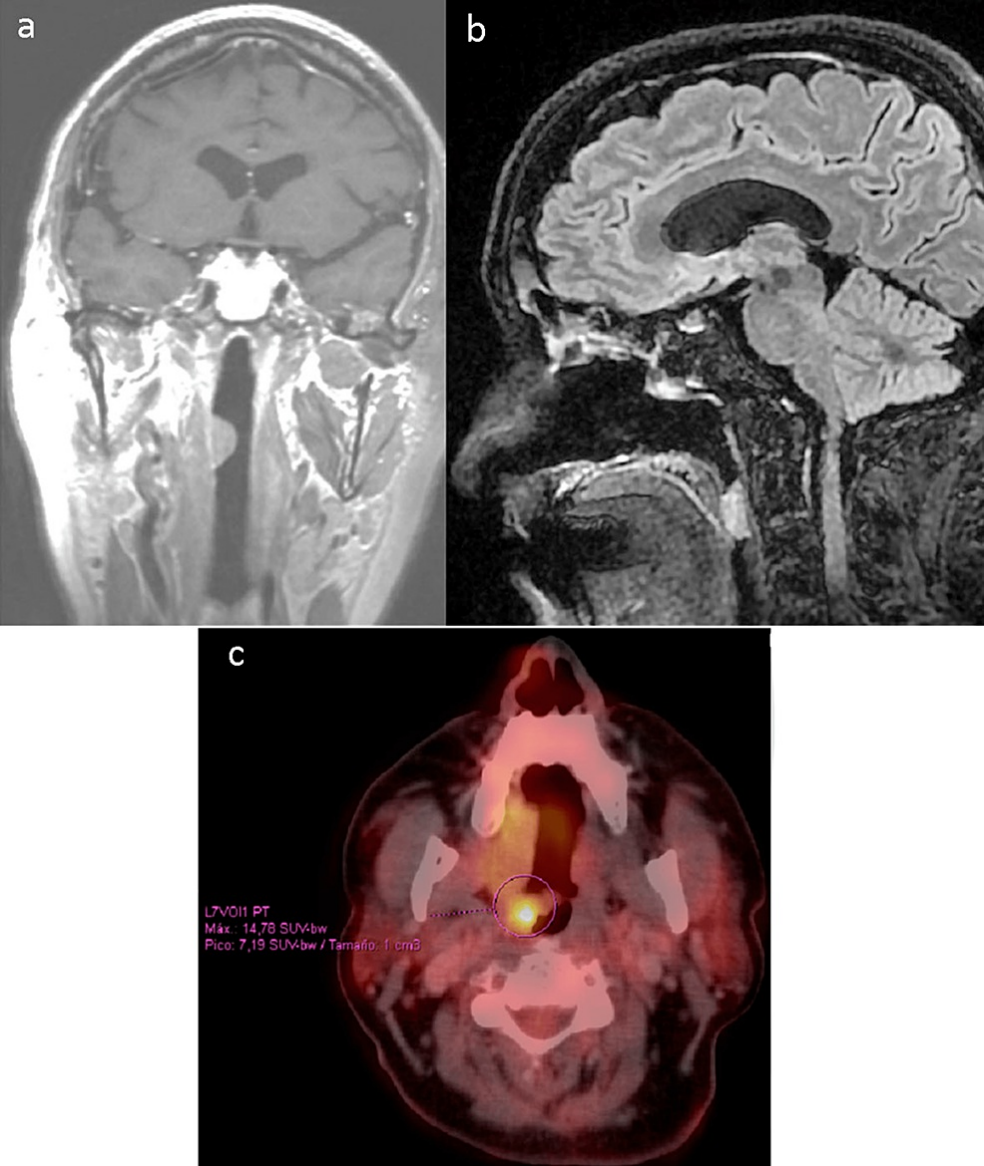
MRI images showing a right oropharyngeal lesion partially behind the soft palate (frontal a, sagittal b). PET-CT with isolated metabolic activity (axial c). PET: Positron emission tomography.

A 77-year-old woman, who had been treated for sinonasal NEN by surgery and chemoradiotherapy at another institution 20 years prior, presented with a history of the condition. She was referred to our service for an annual follow-up due to lacrimal obstruction secondary to the treatment of her previous tumor. She came before her routine follow-up with a complaint of three months of right pharyngeal discomfort without any other accompanying local or systemic symptoms. The endoscopic examination revealed a well-defined lesion on the lateral wall of the oropharynx, partially behind the soft palate, with an even surface. The rest of the ear, nose, and throat (ENT) physical examination was unremarkable, with no other mucosal lesions or cervical masses. The MRI exam revealed the presence of an isolated lesion in the lateral wall of the pharynx (Figure 1a), partially behind the soft palate (Figure 1b). The fluorodeoxyglucose (FDG)-positron emission tomography (PET)-CT scan demonstrated an isolated lesion with a maximum Standard Uptake Value (SUVmax) of 14,78 (Figure 1c) without evidence of sinonasal recurrence or any other site of activity.

**Figure 2:**
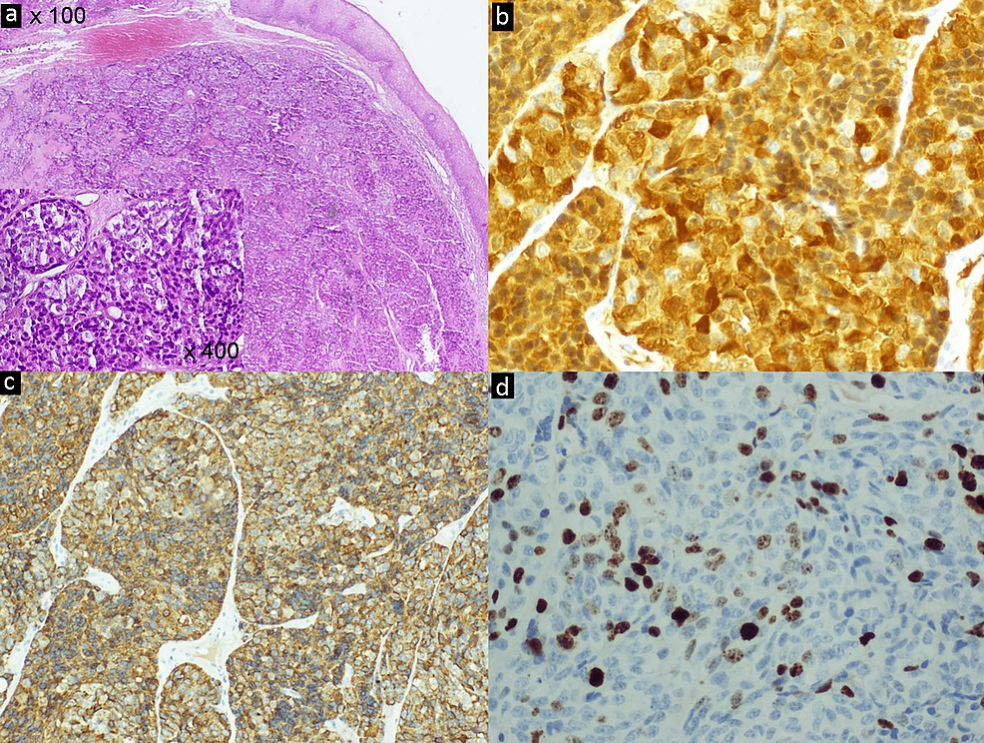
Small cell tumor isolated from the mucosal surface. (a) -HE x100 x400-, positive immunohistochemical staining for chromogranin, (b) -x200- and synaptophysin, (c) -x200-, Ki67 index of 30% of the cells, (d) -x200-.

The patient underwent transoral surgery with radiofrequency, with complete excision of the lesion. The anatomopathological study was consistent with a NEN. The histological examination demonstrated the characteristic image of a tumor with well-defined borders located in the lamina propria with no connection to the mucosal epithelium. It comprised solid nests of epithelial cells with large vesicular nuclei, occasional nucleoli, and clear cytoplasm (Figure 2a), isolated mitoses (1 per mm2), and no evidence of necrosis. The neoplastic cells showed immunoreactivity for characteristic neuroendocrine markers, chromogranin A (Figure 2b), and synaptophysin (Figure 2c). The Ki67 proliferation marker index of 30% classified the tumor as a grade 3 NEN (Figure 2d).

## Discussion

NENs are a heterogeneous group of entities, difficult to classify. NEN refers to a group of well-differentiated neoplasms whose potential for metastasis or local invasion depends on its location, type, and degree (G1 to G3, defined by Ki67 index and mitotic activity). Neuroendocrine carcinoma (NEC) corresponds to lesions with a high degree of histological malignancy (necrosis, high mitosis count) and aggressive biological behavior [1]. We consider our case to be a second metachronous tumor with no anatomical proximity or metabolic activity and no temporal relationship with late appearance, atypical in high-grade NEC [5], and not described for NENs. NENs of the oropharynx are very rare entities that are generally isolated and non-secreting and hence do not typically present systemic symptoms. The lack of specific symptoms and idle biological behavior may delay the diagnosis for years. According to the literature, the improved access to diagnosis by imaging and endoscopy seems to have led to an earlier diagnosis and, therefore, an increase in more localized cases without metastases at the time of detection [6]. As infrequent entities, references to NEN of the head and neck in the literature include isolated cases or very limited series, most of them referring to NECs [4,7]. While treatment recommendations for this condition are not well established, multidisciplinary interventions, including surgery, chemo, and radiotherapy, are often suggested. However, adjunctive treatments are not considered necessary in localized tumors where complete surgical excision is possible. We believe that the communication of each clinical case that is diagnosed and treated will help to improve the therapeutic indications and monitoring of this pathology.

## Conclusions

Isolated NENs are rare and relatively unknown entities with limited references in the literature. These heterogeneous lesions exhibit atypical and varied behaviors that warrant consideration. Single tumors appear as non-specific lesions; only histologic and immunohistochemical studies will give us the definitive diagnosis. The publication of new cases must contribute to the knowledge of these lesions and their treatment and monitoring. The published data recommend surgical treatment in isolated tumors, of which we can ensure a complete resection with no need for complementary chemo or radiotherapy. Although we have not been able to find any publication on a second tumor in NENs, it seems advisable to maintain a long-term follow-up of these patients, even in apparently disease-free cases.
